# Dental Contact Lenses: An Ecological Study of Online Searches in Brazil

**DOI:** 10.1111/idh.12912

**Published:** 2025-04-07

**Authors:** Humberto Alexander Baca Juárez, Paulo Ricardo Martins‐Filho, Juliana Luongo Pelufo, Raphael Silva Bacelo, Frederico Pereira Castilho, Jullia Melo de Menezes, Aline Nachtigall Milbrath, Carolina Rodriguez Garralaga, Émely Regina Fila, Tatiana Serutina Lopes Da Silva, Francisco Wilker Mustafa Gomes Muniz

**Affiliations:** ^1^ Department of Periodontology Federal University of Pelotas Pelotas Brazil; ^2^ Graduate Program in Health Sciences and Graduate Program in Dentistry Federal University of Sergipe Lagarto Brazil; ^3^ School of Dentistry, Federal University of Pelotas Pelotas Brazil

**Keywords:** dental esthetics, dental research, population characteristics

## Abstract

**Objective:**

The study aimed to investigate trends in searches for “dental contact lenses” on Google Trends and their association with sociodemographic variables of Brazilian states.

**Methods:**

This ecological study utilised Google Trends to analyse search frequencies for “dental contact lenses” in Brazilian states over 5 years. Data were collected from two categories: “health” and “all categories”. Exploratory variables included total population, per capita income, Gini index, illiteracy rate, use of internet, number of dentists, number of specialists in Periodontology, Operative Dentistry and Dental Prosthesis, number of dental schools, and healthcare units. Pearson correlations and time series analyses were conducted.

**Results:**

São Paulo, Santa Catarina, the Federal District, and Tocantins exhibited the highest search frequencies in the “Health” category, while Minas Gerais had the highest search when considering all categories. Some states in the Northeast demonstrated the lowest search frequencies in both categories. Significant and positive correlations were observed between the outcome, when using all categories, and the following variables: total population (*r* = 0.500), per capita income (*r* = 0.468), use of internet (*r* = 0.522), higher education (*r* = 0.686), and number of dentists (*r* = 0.806). Inverse correlations were detected with Gini index (*r* = −0.467), illiteracy rate (*r* = −0.734), number of public dental schools (*r* = −0.430), and primary health units (*r* = −0.472). A similar pattern of results was observed for the health category across most exploratory variables. Both analyses revealed a pattern of seasonality (*p* < 0.001).

**Conclusion:**

Higher socio‐economic status and concentrations of dental professionals in the Brazilian states resulted in higher searches for “dental contact lenses.”

## 1 Introduction

Several dental materials are available in the market and are frequently selected for the restoration of anterior teeth [1]. Dental contact lenses constitute a cosmetic approach in which a thin layer of ceramic or composite resin is applied to the coronal portion of the tooth. This procedure is designed to enhance the aesthetic aspect of an individual's smile, addressing issues such as tooth stains, discolouration, irregular alignment, and gaps [2].

The interest in dental ceramics has been increasing, driven by the growing demand for enhancing dental aesthetics [3]. This trend is further elucidated by recent advancements in technologies and methodologies specifically tailored for the field of operative dentistry [4]. However, no study has yet assessed the public interest in this oral health treatment and explored potential associated variables.

Google Trends provides unique and comprehensive analytics regarding the relative frequency of web searches within the Google database on a broad scale. Since its inception in 2012, Google Trends has offered valuable information about medical research. It has proven to be useful as a surveillance tool for monitoring pandemics and has also served as a substitute for gauging public interest in traditional medical interventions [5]. This platform allows the monitoring of fluctuations in the volume of Google searches for specific keywords over time [6].

Many of these searches belong to health areas, symptoms, management, emergencies, and disease treatments. A study that investigated searches during the early months of the pandemic, using terms such as ‘dentistry,’ ‘coronavirus,’ ‘COVID‐19,’ ‘SARS‐CoV‐2,’ and ‘pandemic,’ revealed that these searches reached their peak before new cases of COVID‐19 were reported. This data demonstrates the importance of examining online interest in these terms, as significant positive correlations were observed between the number of new cases and Google Trends data [6] in several countries, including China, South Korea, Italy, and Germany [7].

Furthermore, it is crucial to understand the frequency with which individuals search for health‐related information, particularly in the context of oral health. These estimates can potentially affect the demand for specific dental treatments and influence decisions regarding dental procedures. Therefore, this study aimed to analyse searches for the term ‘dental contact lenses’ on Google Trends across all states in Brazil and their association with several exploratory variables. The working hypothesis is that there would be different patterns of searches related to “dental contact lenses” among the different Brazilian states, including a positive correlation between searches and greater socio‐economic conditions and higher concentrations of dental professionals.

## 2 Methods

### 2.1 Study Design

This is an ecological study that used publicly available data from all Brazilian states. As a result, ethical approval was not deemed necessary for this study. This study followed the STROBE checklist.

### 2.2 Data Collection—Outcome

Google Trends database was used to obtain relative search frequencies for the term “dental contact lenses” (search term in Portuguese: “lentes de contato dental”) in each Brazilian state. The presented data were anonymised, categorised, and aggregated on a scale of 0–100. The state with the highest number of searches was identified as the peak, indicating sudden spikes in search interest, while other states received proportional frequencies. The data were extracted from Google Trends on July 15th, 2023, and the data extraction was limited to Brazil, covering the past 5 years. Two distinct data collections were conducted, one focused on the “health area” and another encompassing “all categories.” This dual approach allowed for a comprehensive view of the search trends, differentiating between health‐specific interest and a broader spectrum of queries potentially including aesthetic or general curiosity factors.

### 2.3 Data Collection—Exploratory Variables

Several pieces of information were obtained from the Brazilian Institute of Geography and Statistics (Instituto Brasileiro de Geografia e Estatística [IBGE] in Portuguese), based on the 2022 Brazilian census [8]. For each Brazilian state, the collected variables were the following: total population, number of males and females, per capita income, number of individuals with completed high school education, and number of individuals categorised as white, black, or brown (note that data for yellow and Indigenous individuals were not available in this database). Additionally, the illiteracy rate was gathered. Moreover, for each state, the 2010 Gini index data [9], which is the most recent available, along with the rate of internet access, were extracted from the IBGE website [10].

The number of dentists was sourced from the Federal Council of Dentistry (Conselho Federal de Odontologia [CFO] in Portuguese). Additionally, on the CFO website, data on the number of registered specialists in Operative Dentistry, Periodontology, and Dental Prosthesis were also collected [11]. This data collection occurred on July 15th, 2023.

Information regarding the number of dental schools was collected from the Ministry of Education (Ministério da Educação [MEC] in Portuguese). Specifically, data on the total number of dental schools, number of public (federal, state, and municipal) dental schools, as well as private dental schools (both those with and without lucrative objectives) were extracted from the MEC website [12].

Information regarding healthcare services and centres was sourced from the Brazilian government's digital platform [13]. This data included variables such as the number of Primary Health Care (with or without an oral health team) and Secondary Oral Health Care units [14, 15]. The percentage of population coverage by oral health teams in the primary health care by state was also collected, and this information was taken from the government website [16]. For this variable, data was gathered between 2019 (same starting point of the outcomes) and 2021 (last data available in the website).

### 2.4 Statistical Analysis

The primary outcomes of this study were the relative frequencies of the term “dental contact lenses” in both all categories and the health‐related category. Data were organised into an Excel spreadsheet and analysed by SPSS software (version 29.0 for MAC). The unit of analysis for this study was the Brazilian states.

Pearson correlations were performed for each exploratory variable. These variables were analysed as follows:

- Total population (in millions);
- Per capita income;
- Gini index;
- Illiteracy rate;
- Percent of use of the internet;
- Proportion of females to males;
- Proportion of white to non‐white individuals (non‐white individuals included those who identified as black or brown);
- Number of individuals with complete high level of education divided by millions of population;
- Total number of dental schools divided by millions of population;
- Number of public dental schools divided by millions of population;
- Number of private dental schools divided by millions of population;
- Number of Primary Health Care units (with or without oral health teams) divided by millions of population.
- Number of Secondary Oral Health Care Units divided by millions of population.
- Percentage of population coverage by oral health teams in the primary health care.
- Number of dentists divided by millions of population;
- Number of registered specialists in Operative Dentistry, Periodontology, and Dental Prosthesis divided by millions of population.

For all analyses, statistical significance was established at *p* < 0.05. As demonstrated in the literature [17], correlations are interpreted as perfect (correlation coefficient of +1 or −1), very strong (correlation coefficient ranging from +0.9 to +0.8 or from −0.9 to −0.8), moderate (correlation coefficient ranging from +0.7 to +0.6 or from −0.7 to −0.6), fair (correlation coefficient ranging from +0.5 to +0.3 or from −0.5 to −0.3), poor (correlation coefficient ranging from +0.2 to +0.1 or from −0.2 to −0.1), or none (correlation coefficient of 0). Due to the low number of units of analysis, linear regression analysis was not feasible.

Moreover, time series analyses were conducted using the “ggplot2,” “forecast,” and “tseries” packages in the *R* software. For each outcome, data were aggregated into an annual frequency (comprising 52 weeks), and time series decomposition was performed, illustrating trends, seasonality, and residuals through graphical representations. The Ljung‐Box test was employed to assess the independence of the residuals in the time series model.

## 3 Results

In the “health” category, it was shown that the states of São Paulo (100), Santa Catarina (97), the Federal District (92), and Tocantins (92) exhibited the highest frequencies among all the states of Brazil, whereas Ceará (65), Paraíba (65), Rio Grande do Norte (65), and Sergipe (60) demonstrated the lowest frequencies (Figure S1). When conducting the same search for “all categories,” it was observed that Minas Gerais (100) had the highest number of searches, followed by São Paulo (98), the Federal District (91), and Santa Catarina (91). Conversely, the lowest search volumes were also observed in states located in the Northeast of Brazil, including Alagoas (64), Ceará (63), Rio Grande do Norte (62), and Sergipe (61) (Figure S2). For both outcomes, there was either insufficient information or unavailable data to quantify the search trends for the following states: Acre, Amapá, and Roraima (Figures S1 and S2).

Table 1 presents the correlation analyses between searches for “dental contact lens” and the exploratory variables. When considering all categories of search, a very strong and positive correlation was identified only with number of dentists. Other significant and positive correlations were also identified, as moderate (number of individuals with complete higher education and number of specialists in Periodontology) and fair (total population, per capita income, rates of use of internet, number of specialists in Operative Dentistry, and number of specialists in Dental Prosthesis). For the health category, only moderate (per capita income, number of individuals with complete higher education, number of dentists, and number of specialists in Periodontology) and fair (total population, rates of use of internet, number of specialists in Operative Dentistry, and number of specialists in Dental Prosthesis) positive correlations were detected. Conversely, negative and moderate correlations were identified to illiteracy rate to both outcomes. Fair and negative correlations were found for the Gini index, population coverage by oral health teams in the primary health care, number of public dental schools, and number of Primary Health Care Units.

**TABLE 1:** Correlation between the interest in the term “dental contact lens” (in Portuguese) and exploratory variables of each Brazilian state, based on all categories and health.

	5 years (all categories) *R* ^a^ (*p*‐value)	5 years (health) *R* ^a^ (*p*‐value)
Total population (in million)	**0.500 (*p* = 0.013)**	**0.443 (*p* = 0.030)**
Per capita income (per thousand)	**0.468 (*p* = 0.021)**	**0.729 (*p* < 0.001)**
Gini index	**−0.467 (*p* = 0.021)**	**−0.522 (*p* = 0.009)**
Illiteracy rate	**−0.734 (*p* < 0.001)**	**−0.743 (*p* < 0.001)**
Use of internet	**0.522 (*p* = 0.009)**	**0.469 (*p* = 0.021)**
Proportion female/Male in 2022	−0.246 (*p* = 0.248)	−0.234 (*p* = 0.271)
Proportion white/Non‐white in 2022	0.311 (*p* = 0.13**9)**	**0.492 (*p* = 0.015)**
Complete high level of education (in thousands)^b^	**0.686 (*p* < 0.001)**	**0.700 (*p* < 0.001)**
Total School of Dentistry^b^	0.331 (*p* = 0.114)	0.278 (*p* = 0.189)
Public School of Dentistry^b^	**−0.430 (*p* = 0.036)**	**−0.542 (*p* = 0.006)**
Private School of Dentistry^b^	0.404 (*p* = 0.050)	0.375 (*p* = 0.071)
Primary Health Care Units^b^	**−0.472 (*p* = 0.020)**	**−0.509 (*p* = 0.011)**
Population coverage by oral health teams in the primary health care (in %)	−0.398 (*p* = 0.054)	**−0.430 (*p* = 0.036)**
Secondary Oral Health Care Units^b^	−0.312 (*p* = 0.137)	**−0.425 (*p* = 0.038)**
Number of dentists^b^	**0.806 (*p* < 0.001)**	**0.779 (*p* < 0.001)**
Number of specialists in Operative Dentistry^b^	**0.519 (*p* = 0.009)**	**0.535 (*p* = 0.007)**
Number of specialists in Periodontology^b^	**0.661 (*p* < 0.001)**	**0.605 (*p* = 0.002)**
Number of specialists in Dental Prosthesis^b^	**0.531 (*p* = 0.008)**	**0.507 (*p* = 0.011)**

^a^ Pearson correlation.

^b^ Variable is a proportion divided by a million of the state population. Bold values mean statistically significant correlation (*p* < 0.05).

The proportion of white to non‐white individuals showed a significant positive fair correlation with searches for “dental contact lens” when only the health category was taken into account (*r* = 0.492; *p* = 0.015), indicating the states with higher proportion of white individuals presented higher searchers. Furthermore, the number of Secondary Oral Health Care Units exhibited a significant negative fair correlation with the outcome only within the health category (*r* = −0.425; *p* = 0.038).

Figure S3 displays the results of the time series analyses conducted for each outcome. In both analyses, a pattern of seasonality was demonstrated (*p* < 0.001), occurring in the following periods: from March to mid‐April, the last week of June, and in late October to early November.

## 4 Discussion

This study aimed to investigate the trends and associated exploratory variables related to searches for “dental contact lenses” among all Brazilian states. Higher search volumes were observed in states characterised by larger populations, higher incomes, and a greater number of dentists, regardless of the dental specialty. Conversely, lower search frequencies were associated with states exhibiting a higher Gini index and number of public dental schools. In addition, higher variations in search patterns were observed, which can be interpreted in the context of socio‐economic disparities and awareness of advanced dental procedures. Whenever feasible, data were standardised to the total population to provide more precise estimates.

States in the Northeastern region of Brazil exhibit the lowest search rates, reflecting their reputation for having the country's lowest socio‐economic scores. These findings illuminate the factors contributing to disparities in dental service utilisation, underscoring how healthcare investments and the scarcity of dental professionals in these areas affect individuals' access to oral care information [18]. Conversely, states with better economic standings, such as São Paulo, Santa Catarina, and the Federal District, potentially lead to broader accessibility to oral health information and services. It is worth noting that aesthetic treatments require specific conditions for implementation [19], possibly explaining the heightened frequency of searches detected in these states. However, it must be highlighted that the search trends performed in the present study may not predict the search for oral health treatments.

When interpreting the present results, it is crucial to consider self‐perception of oral health as a potential influencing factor among residents of the Northeast region of Brazil. This positive self‐perception was observed in approximately one‐third of this population, which was linked to several factors, such as demographical, economical, and oral health characteristics [20]. Moreover, some variables can impact the likelihood of not seeking oral treatment. For instance, men and older individuals appear to be more prone to never visiting a dentist, whereas white individuals tend to be slightly more likely to make dentist appointments [21, 22]. Having health insurance, living in urban areas, having more years of education, and a higher income are also associated with an increased likelihood of seeking dental treatment, which corroborates with the results of the present study. However, it is important to note that the availability of dental services varies by location, with underserved areas generally having limited access to dental care [21].

It may be speculated that individuals with higher socio‐economic status tend to adopt new technologies and innovations more rapidly, which include advancements in dental care. For instance, research indicates that regions with a higher concentration of dental professionals are more aware of advanced dental treatments, which may include dental contact lenses [23].

Nonetheless, the negative correlation observed between the Gini index (fair correlation), the number of public dental schools (fair correlation), and the illiteracy rate (moderate correlation), in relation to online searches, implies significant disparities in access to dental health information. These socio‐economic inequalities can restrict access to knowledge about advanced dental procedures, such as dental contact lenses, within less privileged communities [24].

Moreover, this study highlights the need of addressing health disparities in Brazil, especially when considering that the treatment with veneers is expensive. Additionally, it is noteworthy that such procedures typically fall outside the coverage of the Brazilian public healthcare system. In this context, people in states with higher income inequality might be less likely to seek information or services that are perceived as non‐essential or luxurious due to economic constraints. Thus, socio‐economic and educational disparities directly affect awareness and, potentially, access to advanced dental care. Prioritising the promotion of equitable oral health requires targeted efforts to enhance access to education, income, and healthcare services in less privileged regions [21].

The current results indicate consistent trends across both the general and health categories in the Google Trends database, with some exceptions, particularly regarding the variables of racial demographics and the number of Secondary Oral Healthcare Units observed when considering the health category. It is worth noting that these centres are required to house specialties like periodontology, endodontics, special needs dentistry, and oral and maxillofacial disciplines, but not operative dentistry and dental prosthesis, which are more aligned with aesthetic treatments. This mandatory specialisation might elucidate the observed results.

In both of the searches conducted, patterns of seasonality were identified. These patterns emerged in March through mid‐April, reappeared in the last week of June, and recurred in late October and early November. Notably, in Brazil, Dentist Day is celebrated on October 25th. This observation leads to a speculation that there is a heightened level of media attention directed towards oral health professionals during this specific period, which could account for the increased interest in oral health procedures. Furthermore, it is a widely held belief that patients tend to seek these procedures more frequently in anticipation of the year‐end holidays. This anticipation aligns with the observed surge in searches during late October and early November.

The limitations encountered throughout this study can be attributed to its ecological nature. In such studies, one depends on the associations and relationships within secondary data, and it is not possible to ascertain the direct reasons driving people to search for dental contact lenses—whether it is for aesthetic or health‐related reasons. In this type of study, the data under analysis is usually of a secondary nature. Ecological studies are valuable for generating prompt responses and hypotheses. However, they may not always serve as suitable tools for hypothesis testing due to certain inherent limitations [25].

This study elucidates the regional disparities in Google search trends for “dental contact lenses” in Brazil, highlighting a correlation with socio‐economic indicators and the density of dental professionals. The findings indicate a predilection for such searches regions with greater economic affluence and a denser concentration of dental practitioners, contrasting with lower trends in regions with a high number of public dental schools and increased illiteracy rates. Further investigations should probe the implications of these trends on health policy and clinical practice, potentially facilitating targeted interventions to enhance the accessibility of aesthetic dental treatments and align public dental education with emerging interests and market demands. This could foster a more informed and adaptive approach to oral healthcare strategy and delivery in Brazil.

## 5 Conclusion

This study concluded that, among the Brazilian states, increased socio‐economic status and a higher concentration of dental professionals correlated with elevated searches for “dental contact lenses” on Google database.

## 6 Clinical Relevance

### 6.1 Scientific Rationale for Study

Understanding how often people seek information related to health, especially regarding oral health, is essential.

### 6.2 Principal Findings

This study showed variations in Google search patterns for “dental contact lenses” across different regions in Brazil, demonstrating that higher socio‐economic factors and concentration of dental professionals were correlated with higher searchers in the term.

### 6.3 Practical Implications

Socioeconomic disparities may limit the availability of information on advanced dental procedures, such as dental contact lenses.

## Author Contributions

H.A.B.J. and P.R.M.F., and F.W.M.G.M. designed the study. H.A.B.J., J.L.P., R.S.B., J.M.M., F.P.C., A.N.M., C.R.G., É.R.F., and T.S.L.D.S. collected the data. F.W.M.G.M. and P.R.M.F. analysed the data. H.A.B.J. validated the results. H.A.B.J., J.L.P., R.S.B., and J.M.M. wrote the manuscript. All authors revised the literature, corrected the manuscript, and approved its final version.

## Conflicts of Interest

The authors declare no conflicts of interest.

## Supporting information


Figure S1.

## Data Availability

The data that support the findings of this study are available from the corresponding author upon reasonable request.
