# Supplementary material for: Dental Contact Lenses: An Ecological Study of Online Searches in Brazil
Source: Int J Dent Hyg. 2025 Apr 7;23(4):637–42. doi: 10.1111/idh.12912 (PMC12515991; doi:10.1111/idh.12912)
Supplement: Supplementary file 1 — Figure S1. [file IDH-23-637-s001.docx]

**ONLINE APPENDICES**

**Dental contact lenses: an ecological study of online searches in Brazil**

Humberto Alexander Baca Juarez, Paulo Ricardo Martins-Filho, Juliana Luongo Pelufo, Raphael Silva Bacelo, Frederico Pereira Castilho, Jullia Melo de Menezes, Aline Nachtigall Milbrath, Carolina Rodriguez Garralaga, Émely Regina Fila, Tatiana Serutina Lopes Da Silva, Francisco Wilker Mustafa Gomes Muniz

**FIGURE LEGENDS**


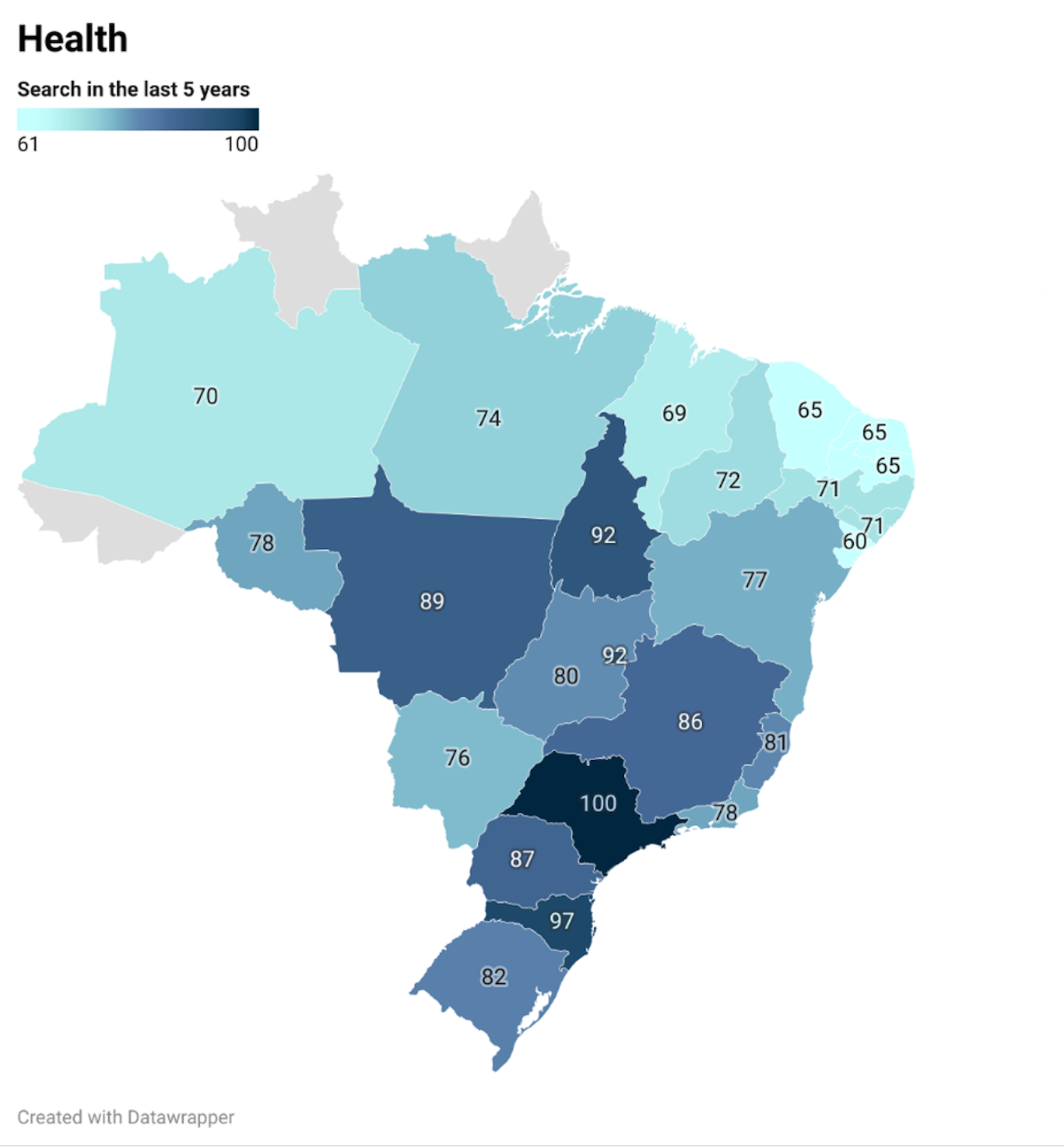


Figure S1. Heat map showing the frequency of searches for the term "dental contact lens" in the category "health" in the last 5 years.


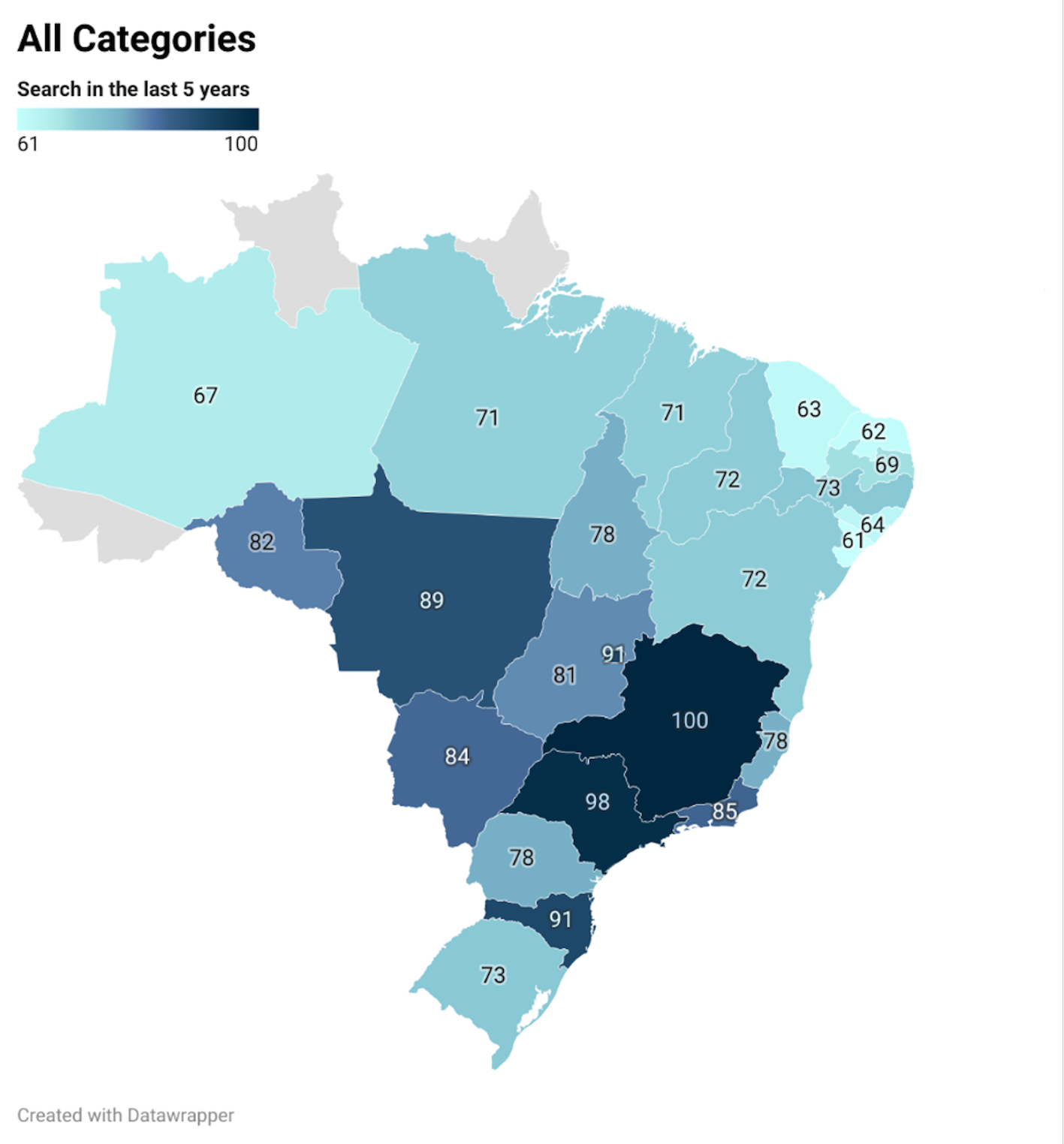


Figure S2. Heat map showing the frequency of searches for the term "dental contact lens" in the category "all categories" in the last 5 years.


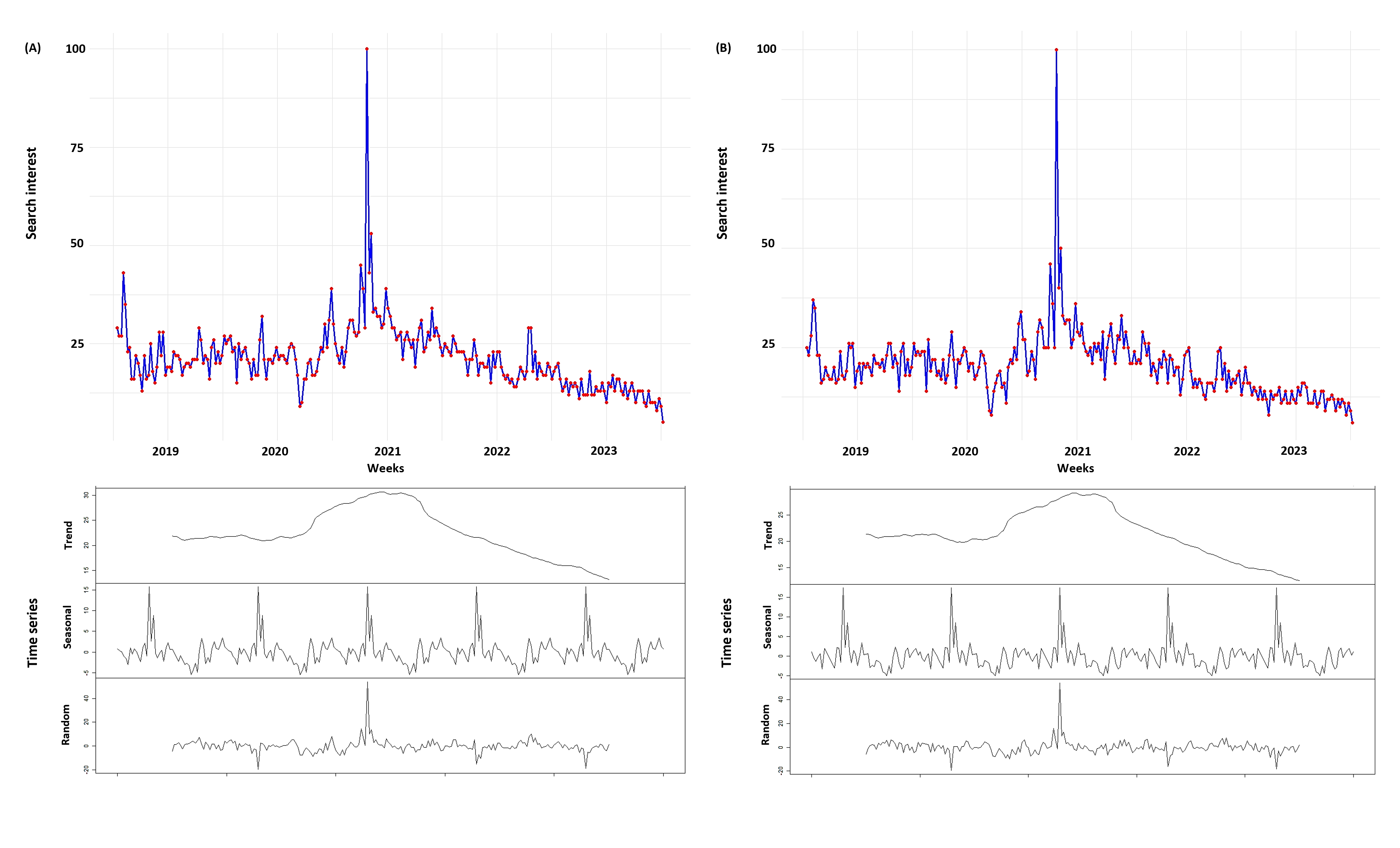


Figure S3. (A) Time series analyses for the search for the term "dental contact lenses" (search term in Portuguese: “lentes de contato dental”) when considering all categories. (B) Time series analyses for the search for the term "dental contact lenses" (search term in Portuguese: “lentes de contato dental”) when considering only the “Health” category. "Trend" shows the underlying trend of the time series. "Seasonal" shows the seasonal component, such as which weeks or months of the year there is a seasonal pattern for the tested outcome. "Random" shows the residual, or the part of the series that cannot be explained by trend or seasonality.
